# Clinical utility of the GeneXpert assay for the diagnosis of *Clostridium difficile* infections

**DOI:** 10.1186/1471-2334-14-S7-O34

**Published:** 2014-10-15

**Authors:** Dragoş Florea, Dan Oțelea, Alexandru Rafila, Ioana Bădicuț, Mona Popoiu, Gabriel Adrian Popescu

**Affiliations:** 1 National Institute for Infectious Diseases "Prof. Dr. Matei Balş", Bucharest, Romania; 2 Carol Davila University of Medicine and Pharmacy, Bucharest, Romania

## Background

Since 2011 a rapid increase of cases with *Clostridium difficile* infection (CDI) was observed in the National Institute for Infectious Diseases (NIID), the largest tertiary level infectious diseases hospital in Romania. The need of a fast and accurate diagnosis of CDI and the low sensitivity of the available rapid immunoassays supported the introduction of a molecular assay as a part of the CDI diagnostic strategy. The present study aimed to evaluate the clinical utility of the GeneXpert *Clostridium difficile* (Cepheid, Sunnyvale, CA) assay in the diagnosis of CDI cases in NIID.

## Methods

Retrospective study of the CDI cases admitted or presented at NIID between January 2013 and August 2014. We used the medical records to analyze the demographic and medical data of each patient, the positivity rate and the time to result for the GeneXpert assay and for the standard solid medium anaerobic cultivation.

## Results

A number of 1454 samples from 1289 patients were tested with GeneXpert in the last 19 months. The mean age in the studied group was 59 years (0-95 years) and the male:female ratio was 1:1.32. Approximately half (49.2%) of the samples tested with the molecular assay were positive for toxigenic *C. difficile*. A presumptive identification of *C. difficile* ribotype 027 was done in most (80.8%) of the positive samples. Due to its sample based format the molecular assay had a much shorter time to result than the standard culture tests (2.47 hours versus 3.86 days).

## Conclusion

Sample based molecular assays are an important tool in the management of CDI, providing valuable information for timely treatment decisions and for appropriate institutional infection control procedures.

